# miR-363 suppresses the proliferation, migration and invasion of clear cell renal cell carcinoma by downregulating S1PR1

**DOI:** 10.1186/s12935-020-01313-9

**Published:** 2020-06-10

**Authors:** Yongpeng Xie, Luyao Chen, Yu Gao, Xin Ma, Weiyang He, Yu Zhang, Fan Zhang, Yang Fan, Liangyou Gu, Pin Li, Xu Zhang, Xin Gou

**Affiliations:** 1 Department of Urology, The First Affiliated Hospital of Chongqing Medical University, No. 1 Youyi Road, Yuzhong District, Chongqing, 400016 China; 2 Department of Urology, State Key Laboratory of Kidney Diseases, Chinese PLA General Hospital, No. 28, Fuxing Road, Haidian District, Beijing, 100853 China; 3 Department of Urology, The First Affiliated Hospital of Nanchang University, Nanchang, Jiangxi China; 4 Department of Pediatric Urology, Bayi Children’s Hospital Affiliated to the Seventh Medical Center of Chinese PLA General Hospital, Beijing, China

**Keywords:** miR-363, Proliferation, Migration, Invasion, S1PR1, Clear cell renal cell carcinoma

## Abstract

**Background:**

MicroRNAs (miRNAs) serve as important regulators of the tumorigenesis and progression of many human cancers. Therefore, we evaluated the biological function and underlying mechanism of miR-363 in clear cell renal cell carcinoma (ccRCC).

**Methods:**

The expression of miR-363 in ccRCC tissues compared with adjacent normal renal tissues was detected by quantitative real-time polymerase chain reaction, and the association between miR-363 levels and prognosis of ccRCC patients was analyzed. The candidate target gene of miR-363 was determined by in silico analysis and luciferase reporter assays. The effects of miR-363 on the proliferation, migration and invasion of ccRCC cells in vitro were determined by MTS assay, colony formation assay, Transwell assay and wound healing assay. We also investigated the roles of miR-363 in vivo by a xenograft tumour model. The mechanism of miR-363 on the proliferation, migration and invasion of ccRCC was determined by gain- and loss-of-function analyses.

**Results:**

we demonstrated that miR-363 expression was obviously downregulated in ccRCC tissues and that reduced miR-363 expression was correlated with poor disease-free survival (DFS) in ccRCC patients after surgery. S1PR1 expression was inversely correlated with the level of miR-363 in human ccRCC samples. Luciferase reporter assays suggested that S1PR1 was a direct functional target of miR-363. miR-363 downregulated S1PR1 expression and suppressed the proliferation, migration and invasion abilities of ccRCC cells in vitro and suppressed xenograft tumour growth in vivo. Importantly, miR-363 exerted its biological function by inhibiting S1PR1 expression in ccRCC cells, leading to the repression of ERK activation. Moreover, we found that the levels of downstream effectors of ERK, including PDGF-A, PDGF-B, and epithelial-mesenchymal transition (EMT)-related genes, were decreased after miR-363 overexpression.

**Conclusions:**

Our results suggest that miR-363 acts as a tumour suppressor by directly targeting S1PR1 in ccRCC and may be a potential new therapeutic target for ccRCC.

## Background

Renal cell carcinoma (RCC) is one of the most common urological malignancies and accounts for approximately 3% of all human malignant neoplasms [1, 2]. The incidence of RCC has steadily increased worldwide, and approximately 30% of patients with a primary diagnosis of RCC have metastases after partial or radical nephrectomy [3, 4]. RCC originates from the renal epithelium and comprises several histological subtypes based on different biological characteristics [5]. Clear cell RCC (ccRCC), the most common and aggressive RCC subtype, is characterized by high rates of local invasion, metastasis and mortality [6, 7]. Although significant improvement in ccRCC treatments, such as targeted therapy, has been achieved in the past two decades, many treated patients will eventually progress to advanced ccRCC with a concomitant poor prognosis [8, 9]. Therefore, a deeper understanding of the mechanisms responsible for ccRCC tumorigenesis and progression and more effective therapies are urgently needed.

MicroRNAs (miRNAs), a class of evolutionarily conserved small non-coding RNAs with lengths of 18–24 nucleotides, can bind to the 3′-untranslated region (3′-UTR) of target mRNAs, leading to inhibition of their translation or degradation [10, 11]. miRNAs are known to contribute to multiple tumorigenic processes in human cancers, including proliferation, apoptosis, migration and invasion [12]. Recently, several studies have reported the differential expression profiles of miRNAs in ccRCC tissues compared to corresponding non-tumour tissues [13–15], indicating an important role for miRNAs in the carcinogenesis and progression of ccRCC. In agreement with our previous miRNA expression profiling results (unpublished), miR-363 is among the most significantly downregulated miRNAs in ccRCC tissues, and this result has been further validated by real-time polymerase chain reaction (PCR). The dysregulation of miR-363 has been found in many malignant tumours, including gastric cancer, prostate cancer, lung cancer and colorectal cancer [16–19]. Nevertheless, to our knowledge, the biological function, prognostic significance and molecular mechanism of miR-363 in ccRCC remain largely unknown.

Sphingosine-1-phosphate receptor 1 (S1PR1) is a member of the G-protein-coupled receptors that engages with sphingosine-1-phosphate (S1P), which is generated by phosphorylation of sphingosine [20]. S1PR1 is a predicted direct target of miR-363 using several algorithms (TargetScan and miRDB), and this is confirmed by luciferase reporter assay. S1PR1 is involved in multiple cellular processes, such as cell proliferation, migration, invasion, vascular maturation and angiogenesis, in many cancers [21]. S1PR1 expression is obviously upregulated in ccRCC tissues, and its expression is inversely correlated with the expression of miR-363. However, the roles of S1PR1 in ccRCC have not previously been explored. In our study, we found that miR-363 was significantly downregulated in ccRCC and that the reduced expression of miR-363 was correlated with poor prognosis. We also demonstrated that miR-363 suppressed the proliferation, migration and invasion abilities of ccRCC cells in vitro and tumorigenic capacity in vivo by directly targeting S1PR1. Finally, we revealed that miR-363 suppressed the malignant phenotype of ccRCC by inhibiting the phosphorylation of extracellular signal-regulated kinase (ERK). These results indicate that miR-363 may serve as a potential tumour suppressor and a therapeutic target in ccRCC.

## Methods

### Patients and tissue samples

A total of 77 ccRCC tissues, paired with adjacent normal tissues, were obtained from patients who underwent nephrectomy in the Department of Urology, Chinese People’s Liberation Army (PLA) General Hospital (Beijing, China). All tissue samples were immediately snap frozen in liquid nitrogen after resection and then maintained at – 80 °C for further use. The pathologic diagnosis of ccRCC for these patients was confirmed by senior pathologists. The TNM stages were determined according to the 2010 American Joint Committee on Cancer (AJCC) classification system, and the nuclear grades were assigned in accordance with the Fuhrman nuclear grading system. Written informed consent was obtained from each enrolled patient, and the experimental procedure was approved by the Ethics Committee of Chinese PLA General Hospital.

### Cell lines and culture

Human ccRCC cell lines 769P, 786O, Caki-1 and SN12-PM6, human renal proximal tubular epithelial cell lines HKC and HK2, and the 293T cell line were purchased from the National Platform of Experimental Cell Resources for Sci-Tech (Beijing, China). The cells were cultured in RPMI–1640 medium (HyClone, USA) or Dulbecco’s modified Eagle’s medium (HyClone, USA) supplemented with 10% foetal bovine serum (Gibco, USA) and maintained in an incubator at 37 °C with 5% CO2.

### Quantitative real-time PCR (qRT-PCR)

The total RNA of tissues and cells was extracted with TRIzol reagent (ComWin Biotech, China). Reverse transcription of mRNA was performed using the TransScript One-Step gDNA Removal and cDNA Synthesis SuperMix Kit (TransGen Biotech, China) based on the manufacturer’s protocols. miRNAs were reverse transcribed with a specific stem-loop RT-PCR. Then, qRT-PCR was conducted with SYBR Green (TransGen Biotech, China) on an Applied Biosystems 7500 Sequence Detection System. Peptidylprolyl isomerase A (PPIA) was used to normalize the relative mRNA expression, and small nucleolar RNA U6 was used to normalize the relative miRNA expression. The relative levels of mRNA and miRNA were calculated using the power formula: 2^−ΔCt^ (ΔCt = Ct_target gene_–Ct_normalizer_). The primer sequences are listed in Additional file 1: Table S1.

### RNAi treatment

The miR-363 mimic, inhibitor, small interfering RNAs (siRNAs) against S1PR1 (siS1PR1) and corresponding negative controls (NCs) were designed and chemically synthesized by GenePharma (Shanghai, China). Transfection was conducted using Lipofectamine^®^ 2000 reagent (Invitrogen, Carlsbad, CA) according to the recommendations of the manufacturer. The necessary experiments were performed 48 h after transfection.

### Western blotting

Total protein from tissues or cells was extracted with RIPA lysis buffer (Solarbio, Beijing, China) containing protease inhibitor (Roche Applied Science, Mannheim, Germany). The protein concentrations were detected by BCA assay. Subsequently, the proteins were separated using 12% SDS-PAGE and transferred to PVDF membranes (Millipore, Billerica, MA). The membranes were blocked with 5% bovine serum albumin (Solarbio, Beijing, China) for 1 h at 37 °C, incubated with specific primary antibodies overnight at 4 °C, and then incubated with horseradish peroxidase-conjugated secondary antibodies for 1 h at room temperature. The signals were visualized using an enhanced chemiluminescence detection reagent (Thermo Fisher Scientific, Waltham, MA), and the target protein expression was normalized to that of β-actin. Rabbit anti-S1PR1 antibody (ab-11424, Abcam), rabbit anti-PDGF-A antibody (ab-203911, Abcam) and rabbit anti-PDGF-B antibody (ab-178409, Abcam) were purchased from Abcam (Cambridge, MA, USA). Rabbit anti-ERK1/2 antibody (#4695, Cell Signaling Technology), rabbit anti-p-ERK1/2 antibody (#9101, Cell Signaling Technology), rabbit anti-E-cadherin antibody (#3195, Cell Signaling Technology), rabbit anti-N-cadherin antibody (#13116, Cell Signaling Technology), rabbit anti-Vimentin antibody (#5741, Cell Signaling Technology) and rabbit anti-ZEB1 antibody (#3396, Cell Signaling Technology) were purchased from Cell Signaling Technology (Danvers, MA, USA). Mouse anti-β-actin antibody (TA-09, ZSGB-BIO), horseradish peroxidase-conjugated goat anti-mouse IgG (ZB-2305, ZSGB-BIO) and goat anti-rabbit IgG (ZB-2301, ZSGB-BIO) antibodies were purchased from ZSGB-BIO (Beijing, China).

### Immunohistochemistry (IHC)

A standard IHC procedure was performed as previously described [22]. The analysis was based on a histoscore containing staining intensity and range scores. The staining intensity was scored on a scale of 0–3 as follows: 0 (negative), 1 (weak staining), 2 (moderate staining) and 3 (strong staining). The staining range was scored according to the percentage of cells stained: 0 (0% staining), 1 (≤ 25% staining), 2 (25–50% staining) and 3 (> 50% staining). Immunostaining analyses were performed independently and blindly by two pathologists.

### Immunofluorescence

The cells were seeded on coverslips 24 h prior to the experiment. After fixation with 4% paraformaldehyde-PBS, the cells were permeabilized with 0.5% Triton X-100 and blocked with 3% bovine serum albumin. Then, the cells were incubated with primary antibody against S1PR1 (ab-11424; Abcam, Cambridge, UK) at a 1:50 dilution. After incubation with rhodamine (TRITC)-conjugated goat anti-rabbit secondary antibodies (ZSGB-BIO, Beijing, China), the nuclei were counterstained with 0.2 mg/mL 4′,6-diamidino-2-phenylindole (DAPI). Images were captured by an Olympus confocal microscope.

### Luciferase reporter assay

The wild-type (WT) or mutated (MUT) 3′-UTR of S1PR1 containing the miR-363 binding site was cloned into a psiCHECK2 dual-luciferase vector (Promega, USA) generated by Genewiz (Beijing, China). 293T cells were co-transfected with a luciferase reporter (WT or MUT) and miR-363 mimics or a negative control using Lipofectamine^®^ 2000 reagent (Invitrogen, Carlsbad, CA). Luciferase activity was measured 48 h after transfection using the Dual-Luciferase^®^ Reporter Assay System (Promega, USA). The relative luciferase activity was calculated on the basis of the firefly luciferase signal normalized to the Renilla luciferase signal in accordance with the manufacturer’s protocol.

### Construction of plasmid and viral infections

For ectopic expression of S1PR1, the full-length fragment of the S1PR1 coding sequence was cloned into the lentiviral vector pLV-EGFP-(2A)-puro (InovoGen Tech. Co., Beijing, China). For ectopic expression of miR-363, the miRNA-363 segment was cloned into pLVshRNA-EGFP(2A)-puro (InovoGen Tech. Co., Beijing, China). The empty vector (EV) was used as a control. The constructed sequence was checked by sequencing. The detailed procedures for viral particle generation and infection and stable transfected cell line selection and establishment were performed as previously reported [23].

### MTS assay

TThe treated cells were seeded in 96-well plates (1000 cells/well) in triplicate. At 0, 24, 48, 72 and 96 after seeding, 20 µl reagent (CellTiter 96^®^ AQueous One Solution, Promega, USA) was added into each well and incubated for another 2 h at 37 °C. Absorbance at 490 nm was measured with a microplate reader (BioTek Instruments, USA). All assays were repeated in triplicate.

### Colony formation assay

The treated cancer cells were seeded into 6-well plates (1000 cells/well) in triplicate. After culturing for 2 weeks, the cells were washed with phosphate buffered saline, fixed with 100% methanol and stained with 1% crystal violet solution. The number of colonies consisting of at least 50 cells was counted. All assays were repeated in triplicate.

### Migratory and invasion assays

Uncoated and Matrigel-coated transwell chambers (Corning, NY, USA) containing polycarbonate membrane filters with a pore size of 8 µm were used to assess the migration and invasive capacity of cancer cells according to the manufacturer’s protocol. A total of 1x10^5^ treated cells in 200 µl serum-free medium were seeded into the upper chamber, and 500 µl medium with 10% FBS was added to the lower chamber. After incubation for 12 h (migration) or 24 h (invasion) at 37 °C, the cells on the upper surface of the chambers were carefully scraped. The cells invading into the lower surface of the chambers were fixed in methanol and stained with 1% crystal violet solution. The cells were counted under a microscope in five random fields. All assays were repeated in triplicate.

### Wound healing assay

A wound healing assay was performed in 6-well plates. The treated cancer cells grown to confluence were serum-starved and scratched by a sterile 200 µl pipette tip and washed with phosphate buffered saline. Images of the same position were captured at 0 and 12 h after scratching. The coverage of the intermediate space was measured at three random positions for each replicate. All assays were performed in triplicate.

### In vivo xenograft tumour growth assay

Animal experiments were approved by the Animal Ethical Committee of Chinese PLA General Hospital and performed in accordance with guidelines for the care and use of laboratory animals and institutional ethical guidelines. A total of 5x10^6^ 786O cells stably transfected with pLV-miR-363 or EV were suspended in 0.1 ml sterilized PBS and then subcutaneously implanted into the left armpit of 4-week-old male BALB/c nude mice (10 mice per group). Tumour volume was measured every week and calculated according to the following formula: V (mm^3^) = 0.5 × length (mm) x width^2^ (mm^2^). The animals were sacrificed for weight measurement, western blot analysis and IHC staining of xenograft tumours 8 weeks after implantation.

### Statistical analysis

SPSS 20.0 (SPSS, Inc., USA) and Prism 6.0 (GraphPad, Inc., USA) software were used for statistical analyses. Normally distributed variables were summarized as the mean ± standard deviation (SD) and analysed by Student’s *t* test. Univariate and multivariate analyses were performed using the Cox proportional hazards model. Disease-free survival (DFS) was used for prognostic analysis, which was defined as the interval from surgery to local recurrence, distant metastasis or death of ccRCC patients. A Cox proportional hazard model and the Kaplan–Meier method were used to assess the significance of miR-363 on DFS. A value of P < 0.05 was considered statistically significant.

## Results

### Differential miR-363 and S1PR1 expression levels in ccRCC and corresponding normal tissues

**Fig. 1:**
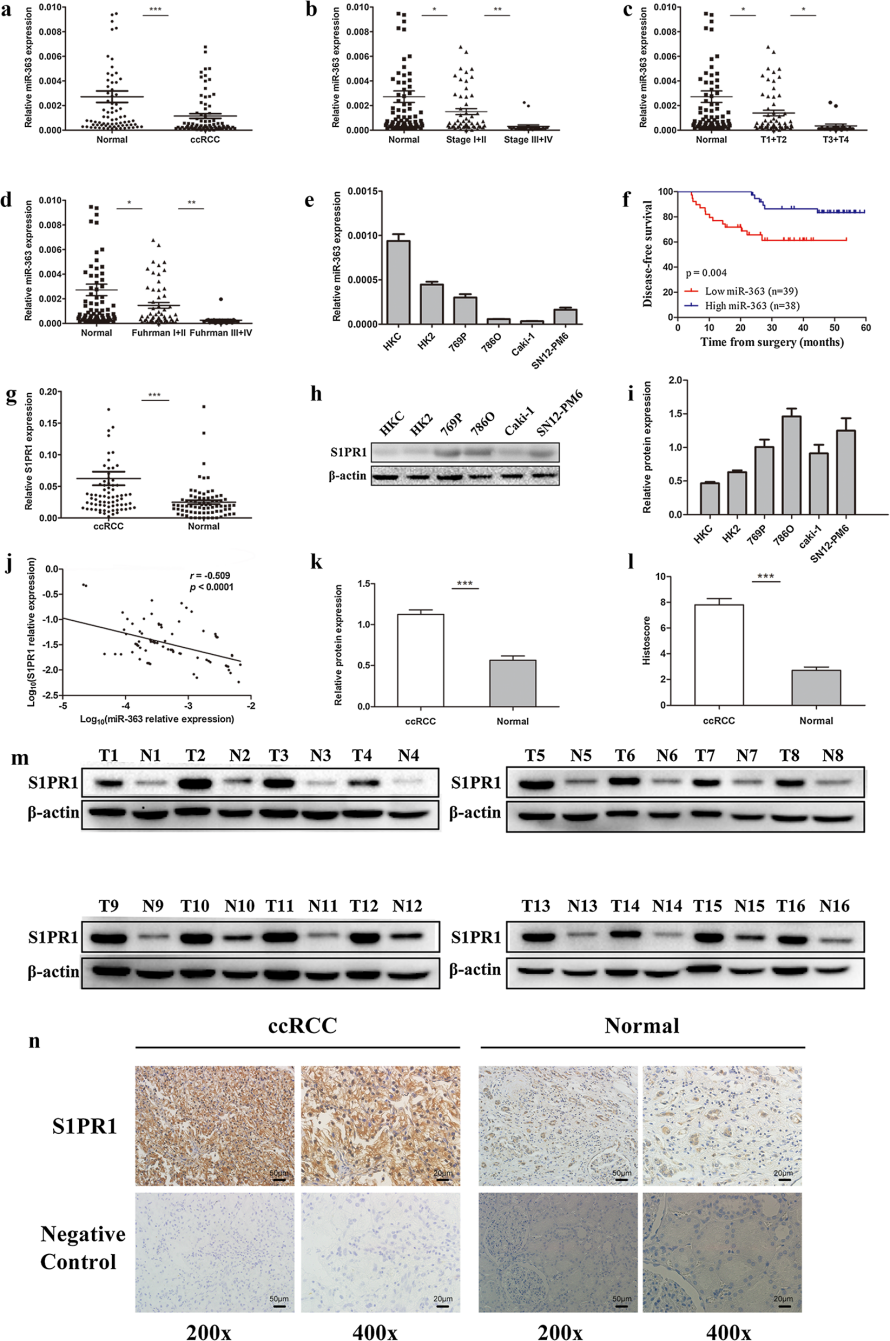
Expression and prognostic significance of miR-363 in ccRCC and its relationship with S1PR1 expression. **a** miR-363 expression levels were significantly downregulated in ccRCC tissues compared to adjacent normal tissues. **b–d** Comparison of miR-363 expression levels between subgroups of patients by clinical stage, T stage and Fuhrman grade. **e** miR-363 expression in normal renal cell lines and various RCC cell lines. **f** Kaplan–Meier analysis of ccRCC patients in the low miR-363 group (n = 39) and the high miR-363 group (n = 38) with regard to disease-free survival. **g** S1PR1 expression levels were markedly upregulated in ccRCC tissues compared to adjacent normal tissues. **h** and **i** S1PR1 protein expression in normal renal cell lines and various RCC cell lines. **j** Negative correlation between S1PR1 mRNA levels and miR-363 levels in ccRCC tissues (n = 77, r = −0.509, P < 0.0001). **k** Quantitative analysis of S1PR1 relative protein expression in ccRCC tissues and adjacent normal tissues by western blot. **l** Histoscores of S1PR1 in ccRCC tissues and adjacent normal tissues based on IHC. **m** Western blot images showed that S1PR1 is upregulated at the protein level in ccRCC tissues, consistent with alterations in mRNA levels in clinical samples. **n** Representative IHC staining images of S1PR1 in ccRCC tissues and their paired normal tissues. Data are presented as the mean ± SD. (*P < 0.05, **P < 0.01, ***P < 0.001)

To validate the miRNA expression profiling results and investigate the role of miR-363 in ccRCC, miR-363 expression was detected in tumour and corresponding normal tissue specimens from 77 ccRCC patients and several cell lines by qRT-PCR. As shown in Fig. 1a, miR-363 was significantly downregulated in ccRCC tissues compared to adjacent normal tissues (P < 0.001). Then, we examined miR-363 expression in the different subgroups of age, sex, Fuhrman grade, T staging, overall TNM staging, microvascular invasion and tumour necrosis of the 77 ccRCC specimens. Relatively low expression of miR-363 was detected in the more developed TNM staging group (P < 0.01, Fig. 1b), the higher T staging group (P < 0.05, Fig. 1c), and the higher Fuhrman grade group (P < 0.01, Fig. 1d). Results from the analysis of the relationship of miR-363 with the clinicopathological features in 77 patients with ccRCC are shown in Table 1. Next, we measured miR-363 expression in multiple cell lines (Fig. 1e). Similar to tissue specimens, miR-363 expression was decreased in ccRCC cell lines (769P, 786O, Caki-1 and SN12-PM6) compared to normal renal cell lines (HKC and HK2). To explore whether miR-363 expression is associated with the prognosis of ccRCC patients, we followed up 77 ccRCC patients for 4.3–59.5 months (median, 35.8 months) after surgery. We selected the median miR-363 expression level as the cut-off value to divide ccRCC patients into low miR-363 group (n = 39) and high miR-363 group (n = 38). Kaplan–Meier analysis demonstrated that patients with low miR-363 expression had poorer DFS (P = 0.004, Fig. 1f). Furthermore, univariate analysis revealed that Overall TNM staging (hazard ratio [HR] = 2.916, 95% confidence interval [CI] 1.190–7.148, P = 0.019) and miR-363 expression (HR = 0.252, 95% CI 0.092–0.691, P = 0.007) were statistically significant predictors of DFS for ccRCC patients. Multivariate analysis using these two factors showed that miR-363 expression (HR = 0.318, 95% CI 0.103–0.983, P = 0.047) was an independent prognostic factor for DFS in patients with ccRCC (Table 2). S1PR1 expression was also detected at the mRNA and protein levels by qRT-PCR and western blotting, respectively. S1PR1 mRNA expression was significantly upregulated in ccRCC tissues compared to adjacent normal tissues (P < 0.001, Fig. 1g). As shown in Fig. 1h, i, the protein expression of SPRR1 was significantly upregulated in ccRCC cell lines (769P, 786O, Caki-1 and SN12-PM6) compared to that in normal renal cell lines (HKC and HK2). Additionally, we also found that there was an inverse relationship between miR-363 and S1PR1 expression at the mRNA level (r = −0.509, P < 0.0001, Fig. 1J). S1PR1 protein expression was also upregulated in ccRCC tissues compared to adjacent normal tissues (P < 0.001, Fig. 1k, m). Immunohistochemistry results showed that positive staining intensity and range were significantly enhanced in ccRCC tissues compared to adjacent normal tissues (P < 0.001, Fig. 1l and n). Overall, these results suggest that miR-363 may serve as a tumour suppressor and that S1PR1 may act as an oncogene in ccRCC.

**Table 1:** The relationship of miR-363 with the clinicopathological features in patients with ccRCC

Clinicopathological features	Number (n = 77)	MiR-363 expression (mean ± SD)	*P* value
Age (years)			
< 60	45	0.00134 ± 0.00169	0.233
≥ 60	32	0.00089 ± 0.00152	
Gender			
Male	65	0.00121 ± 0.00169	0.400
Female	12	0.00078 ± 0.00121	
Fuhrman grade			
I–II	57	0.00146 ± 0.00178	0.004
III–IV	20	0.00026 ± 0.00041	
T staging			
T1–T2	59	0.00139 ± 0.00176	0.016
T3–T4	18	0.00035 ± 0.00064	
Overall TNM staging			
I–II	54	0.00151 ± 0.00180	0.003
III–IV	23	0.00031 ± 0.00057	
Microvascular invasion			
No	58	0.00141 ± 0.00177	0.012
Yes	19	0.00035 ± 0.00063	
Tumor necrosis			
No	49	0.00110 ± 0.00160	0.754
Yes	28	0.00123 ± 0.00170	

*miR‐363* microRNA‐363, *ccRCC* clear cell renal cell carcinoma, *SD* standard deviation, *TNM* tumor node metastasis

**Table 2:** Univariate and multivariate cox regression analysis of clinicopathologic parameters and miR-363 levels with regard to disease-free survival

Variables	Univariate analysis		Multivariate analysis	
	HR (95% CI)	P value	HR (95% CI)	P value
Age (years)				
< 60	Reference			
≥ 60	0.966 (0.394–2.367)	0.940		
Gender				
Male	Reference			
Female	1.925 (0.699–5.304)	0.205		
Fuhrman grade				
I–II	Reference			
III–IV	1.804 (0.682–4.770)	0.234		
T staging				
T1–T2	Reference			
T3–T4	2.051 (0.777–5.412)	0.147		
Microvascular invasion				
No	Reference			
Yes	1.844 (0.700-4.857)	0.215		
Tumor necrosis				
No	Reference			
Yes	1.237 (0.505–3.033)	0.642		
Overall TNM staging				
I–II	Reference		Reference	
III–IV	2.916 (1.190–7.148)	0.019	1.678 (0.616–4.572)	0.311
miR-363 levels				
Low	Reference		Reference	
High	0.252 (0.092–0.691)	0.007	0.318 (0.103–0.983)	0.047

*miR‐363* microRNA‐363, *HR* hazard ratio, *CI* confidence interval, *TNM* tumor node metastasis

### miR-363 inhibits the proliferation, migration and invasion of ccRCC cells in vitro

**Fig. 2:**
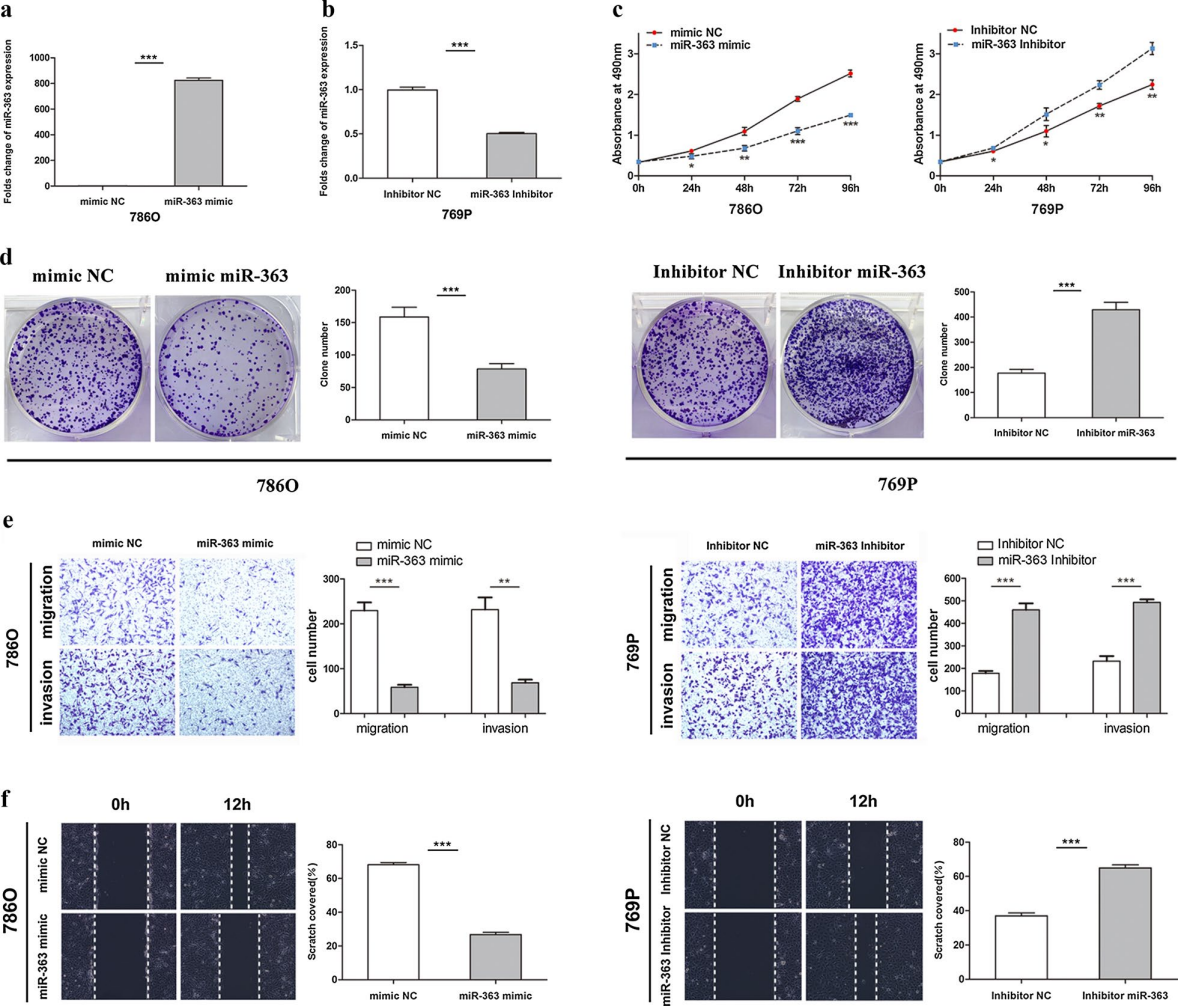
miR-363 inhibits the proliferation, migration and invasion of ccRCC cells in vitro. **a** Folds change of the miR-363 expression levels in 786O cells transfected with miR-363 mimics or NC mimics. **b** Folds change of the miR-363 expression levels in 769P cells with the transfection of the miR-363 inhibitor or NC inhibitor. **c** MTS assay suggested that transfection of the miR-363 mimic inhibited the proliferation of 786O cells and that the miR-363 inhibitor promoted the proliferation of 769P cells. **d** Colony formation assay suggested that overexpression of miR-363 inhibited the colony number in 786O cells, whereas suppression of miR-363 promoted the colony number in 769P cells. **e** Transwell assays suggested that overexpression of miR-363 inhibited migration and invasion in 786O cells and that suppression of miR-363 promoted migration and invasion in 769P cells. **f** Wound healing assay suggested that overexpression of miR-363 inhibited the cell mobility of 786O cells and that suppression of miR-363 promoted the cell mobility of 769P cells. Data are presented as the mean ± SD. (*P < 0.05, **P < 0.01, ***P < 0.001)

To further explore the function of miR-363 in ccRCC, a miR-363 mimic (363 M) was used to elevate the expression of miR-363, and a miR-363 inhibitor (363I) was used to decrease the expression of miR-363 in vitro. 786O cells with relatively low expression of miR-363 were transfected with miR-363 mimic to achieve miR-363 overexpression. The same cells were transfected with miR-363 negative control (NC) mimic as a control (Fig. 2a). Simultaneously, 769P cells with relatively high miR-363 expression were transfected with the miR-363 inhibitor (Fig. 2b). The efficiencies of overexpression and knockdown were verified by qRT-PCR. MTS assays showed that 786O cells transfected with miR-363 mimic had significantly inhibited growth compared to that in the NC mimic group. In contrast, the miR-363 inhibitor significantly enhanced the growth of 769P cells compared to the control cells in the NC inhibitor group (Fig. 2c). Similar results were observed in the colony formation assay: 786O cells transfected with the miR-363 mimic had markedly inhibited colony formation compared with the same cells transfected with the NC mimic. Conversely, 769P cells transfected with miR-363 inhibitor significantly enhanced colony formation ability (Fig. 2d). To investigate the effect of miR-363 on the migration and invasion of ccRCC cells, we performed Transwell and wound healing assays. Transwell assays showed that the migration and invasion abilities were significantly inhibited in miR-363-overexpressing 786O cells compared to miR-363 NC mimic-transfected cells, and inhibition of miR-363 markedly increased the migration and invasion abilities of 769P cells compared to miR-363 NC inhibitor-transfected cells (Fig. 2e). In the wound healing assay, the scratch area recovered at a relatively slower speed in miR-363-overexpressing 786O cells than in miR-363 NC mimic-transfected cells. The scratch area was recovered faster in 769P cells transfected with the miR-363 inhibitor than in those transfected with the miR-363 NC inhibitor (Fig. 2f). Collectively, these results indicated that miR-363 inhibits the proliferation, migration and invasion of ccRCC cells in vitro.

### S1PR1 promotes the proliferation, migration and invasion of ccRCC cells in vitro

**Fig. 3:**
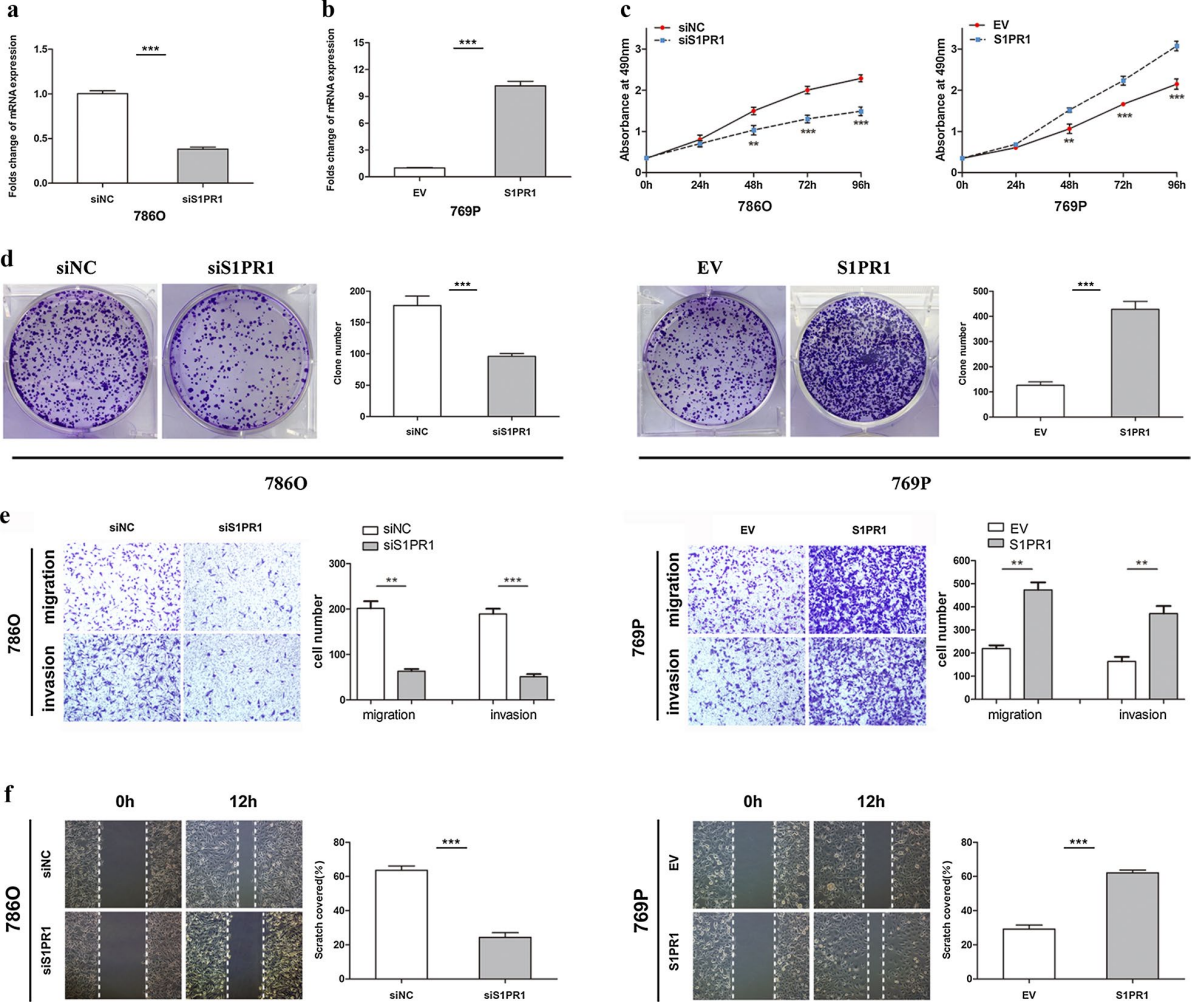
S1PR1 promotes the proliferation, migration and invasion of ccRCC cells in vitro. **a** Folds change of S1PR1 expression levels in 786O cells after knockdown with siRNA. **b** Folds change of the S1PR1 expression levels in 769P cells after overexpression with lentiviral S1PR1 plasmids. **c** MTS assay suggested that knockdown of S1PR1 inhibited the proliferation of 786O cells and that overexpression of S1PR1 promoted the proliferation of 769P cells. **d** Colony formation assay suggested that knockdown of S1PR1 inhibited the colony number in 786O cells and that overexpression of S1PR1 promoted the colony number in 769P cells. **e** Transwell assays suggested that knockdown of S1PR1 inhibits migration and invasion in 786O cells and that overexpression of S1PR1 promoted migration and invasion in 769P cells. **f** Wound healing assay suggested that knockdown of S1PR1 inhibited the cell mobility of 786O cells and that overexpression of S1PR1 promoted the cell mobility of 769P cells. Data are presented as the mean ± SD. (*P < 0.05, **P < 0.01, ***P < 0.001)

It is well known that S1PR1 plays a crucial role in the development and progression of malignant tumours. To explore its biological function in ccRCC, we used the siRNA technique to knockdown S1PR1 and lentiviral particles to achieve S1PR1 overexpression. The efficiencies of knockdown and overexpression were detected by qRT-PCR (Fig. 3a, b). MTS assays illustrated that the proliferation ability was markedly weakened in 786O cells transfected with si-S1PR1 compared to the same cells transfected with siNC. The proliferation ability was remarkably enhanced in S1PR1-overexpressing 769P cells compared with EV group cells (Fig. 3c). In colony formation assays, 786O cells transfected with si-S1PR1 showed significantly inhibited colony formation compared to those transfected with siNC, and overexpression of S1PR1 obviously increased colony formation in 769P cells (Fig. 3d). Transwell assays showed that the migration and invasion abilities were significantly inhibited in S1PR1 knockdown 786O cells compared to those in siNC cells. In contrast, the migration and invasion abilities were markedly increased in S1PR1-overexpressing 769P cells compared with empty vector-group cells (Fig. 3e). In the wound healing assay, the scratch area recovered at a relatively slower speed in S1PR1 knockdown 786O cells than in control cells. Conversely, the scratch area recovered faster in S1PR1-overexpressing 769P cells (Fig. 2f). These results indicated that S1PR1 promotes the proliferation, migration and invasion of ccRCC cells, which suggests that S1PR1 may act as a tumour oncogene in ccRCC.

### S1PR1 is a direct target of miR–363

**Fig. 4:**
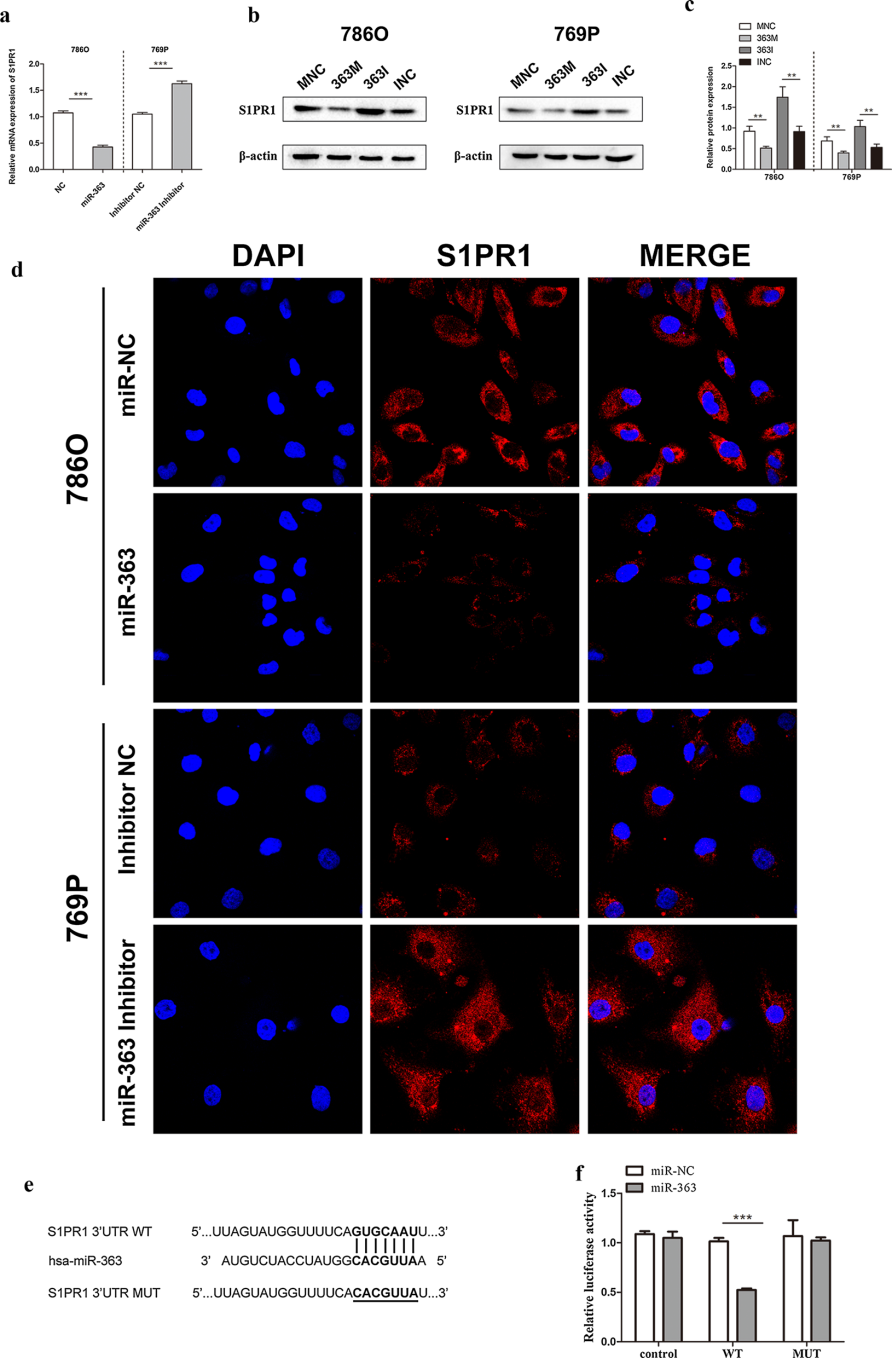
miR-363 downregulates S1PR1 expression by directly targeting its 3′-UTR. **a** S1PR1 mRNA level changes in 786O and 769P cells treated with different interferences. **b**, **c** Overexpression of miR-363 decreased the expression of S1PR1 protein levels in ccRCC cells, whereas knockdown of miR-363 increased the expression of S1PR1 protein levels in ccRCC cells. **d** Representative immunofluorescent staining images showed the inverse effect of miR-363 on S1PR1 in ccRCC cells. **e** Sequence alignment of the S1PR1 3′-UTR with wild-type (WT) versus mutant (MUT) predicted potential miR-363 binding sites. **f** Luciferase reporter assay showed attenuated reporter activity after transfection of the wild-type S1PR1 3′-UTR reporter construct in human embryonic kidney 293T cells overexpressing miR-363. The S1PR1 3′-UTR MUT and control constructs had no effect on reporter activity. Data are presented as the mean ± SD. (*P < 0.05, **P < 0.01, ***P < 0.001)

As the S1PR1 expression level has been previously shown to be elevated in ccRCC tissues and correlated with the progression of clinical stages, we hypothesized that upregulation of miR-363 may inhibit ccRCC malignant progression by attenuating S1PR1 expression. qRT-PCR and western blotting were used to identify the relationship between the expression of miR-363 and S1PR1. As shown in Fig. 4 a–c, S1PR1 mRNA and protein expression levels were significantly decreased in both 786O and 769P cells transfected with miR-363 mimic compared with the respective expression levels in the NC mimic group; however, the miR-363 inhibitor increased S1PR1 mRNA and protein expression in 786O and 769P cells.

In immunofluorescence assays, S1PR1 protein expression was decreased in 786O cells treated with miR-363 mimics compared to cells transfected with NC mimic, and S1PR1 protein expression was increased in 769P cells transfected with the miR-363 inhibitor compared with cells transfected with NC (Fig. 4d). These results reveal that the protein expression of S1PR1 is negatively regulated by miR-363.

Bioinformatics predictions by miRDB (http://mirdb.org/miRDB/) and TargetScan (http://www.targetscan.org/) software validated one conserved miR-363 binding site on the 3′-UTR of S1PR1 mRNA. To investigate whether miR-363 can directly target its binding site on the 3′-UTR of S1PR1 mRNA, we constructed a luciferase reporter in which the 3′-UTR sequence of S1PR1 containing either wild-type or mutant miR-363 putative binding sites was cloned into a luciferase reporter plasmid (Fig. 4e). 293T cells co-transfected with luciferase reporter (wild-type or mutant) or with miR-363 mimic or NC were examined in a luciferase reporter assay. miR-363 overexpression substantially repressed the luciferase activity of the wild-type reporter compared with that of the mutant reporter (Fig. 4f). Overall, these results indicate that S1PR1 is a direct target of miR-363 and negatively regulates S1PR1 expression.

### miR-363 exerts its biological function in vitro by downregulating S1PR1

**Fig. 5:**
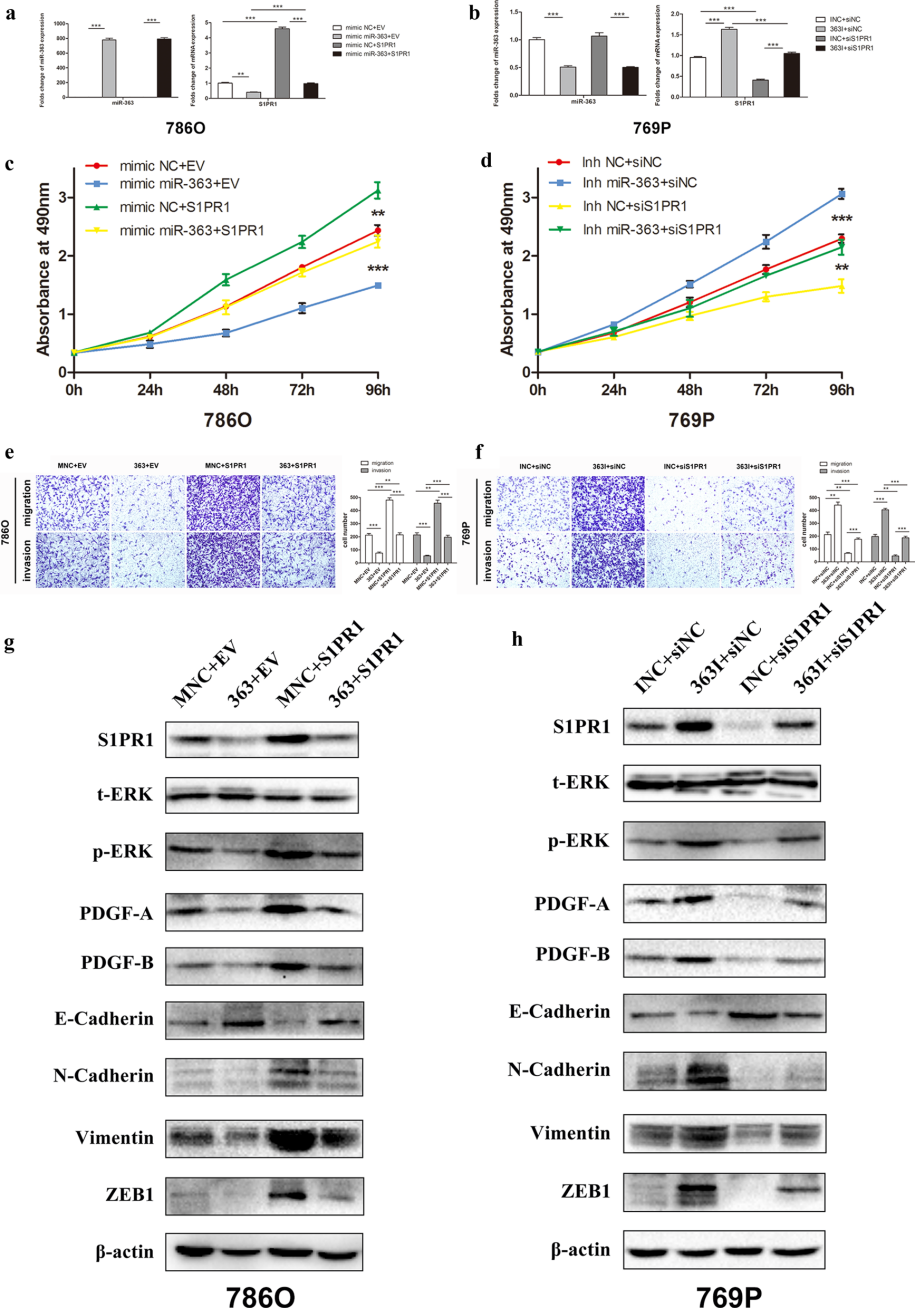
miR-363 inhibits the proliferation, migration and invasion of ccRCC cells by downregulating S1PR1. **a** Folds change of miR-363 and S1PR1 mRNA levels in 786O cells transfected with miR-363 mimic (versus NC mimics) and lentiviral S1PR1 plasmids (versus empty vector). **b** Folds change of miR-363 and S1PR1 mRNA levels in 769P cells transfected with miR-363 inhibitor (versus NC inhibitor) and siS1PR1 (versus siNC). **c** MTS assay suggested that overexpression of S1PR1 reversed the negative proliferative effects of miR-363 mimics in 786O cells. **d** Knockdown of S1PR1 counteracted the positive proliferative effects of miR-363 inhibitor in 769P cells. **e** Transwell assays suggested that overexpression of S1PR1 reversed the negative migration and invasion effects of miR-363 mimics in 786O cells. **f** Knockdown of S1PR1 counteracted the positive migration and invasion effects of miR-363 inhibitor in 769P cells. **g** Alterations of S1PR1, ERK and downstream genes of ERK protein level in 786O cells transfected with miR-363 mimic (versus NC mimics) and lentiviral S1PR1 plasmids (versus empty vector). **h** Alterations of S1PR1, ERK and downstream genes of ERK protein level in 769P cells transfected with miR-363 inhibitor (versus NC inhibitor) and siS1PR1 (versus siNC). Data are presented as the mean ± SD. (*P < 0.05, **P < 0.01, ***P < 0.001)

We examined whether S1PR1 overexpression could reverse the tumour suppressive effects of miR-363 on ccRCC cell proliferation, migration and invasion. First, lentiviral S1PR1 particles or empty vector were co-transfected with miR-363 mimic or mimic NC in 786O cells. qRT-PCR and western blot confirmed that, compared to the mimic NC group, the miR-363 mimic markedly and specifically reduced S1PR1 expression (Fig. 5a and g). Moreover, compared with the inhibitor NC group, the miR-363 inhibitor had significant downregulation of miR–363 and significant upregulation of S1PR1 (Fig. 5b and h). The proliferation, migration and invasion abilities were enhanced in 786O/miR-363 cells after introduction of the lentiviral-S1PR1 particles (Fig. 5c and e). Then, a rescue experiment was conducted by co-transfecting S1PR1 siRNA (versus siNC) and miR-363 inhibitor (versus inhibitor NC) into 769P cells. The reinforced abilities of 769P cell proliferation, migration and invasion caused by the miR-363 inhibitor were effectively reversed by downregulating S1PR1 expression (Fig. 5d and f). These results indicate that miR-363 attenuates the proliferation, migration and invasion abilities of ccRCC cells by downregulating S1PR1.

Then, we investigated the potential biological mechanisms whereby cell proliferation, migration and invasion could be affected by miR-363 in ccRCC. It is accepted that ERK, stimulated by S1PR1, plays important roles in promoting cell proliferation, migration and invasion, so we examined the ERK-related pathways in ccRCC. We found that transfection of miR-363 mimic dramatically inhibited the phosphorylation but not the total levels of ERK, and this effect was reversed by overexpressing S1PR1 in 786O cells (Fig. 5g and Additional file 2: Figure S1a); by contrast, transfection of miR-363 inhibitor markedly promoted the phosphorylation but not the expression of total ERK, and these effects were reversed by downregulating S1PR1 expression in 769P cells (Fig. 5h and Additional file 2: Figure S1b). Subsequently, downstream genes (PDGF-A, PDGF-B and EMT-related genes) of ERK were analysed. We found that transfection of miR-363 mimic dramatically decreased the expression of PDGF-A, PDGF-B, N-cadherin, vimentin and ZEB1 and increased the expression of E-cadherin, which was reversed by overexpressing S1PR1 in 786O cells (Fig. 5g and Additional file 2: Figure S1a); conversely, transfection of miR-363 inhibitor markedly increased the expression of PDGF-A, PDGF-B, N-cadherin, vimentin and ZEB1 and decreased the expression of E-cadherin, which was reversed by downregulating S1PR1 expression in 769P cells (Fig. 5h and Additional file 2: Figure S1b). Overall, the results indicated that miR-363, which inhibited S1PR1 expression, reduced ERK activation and thereby affected cell proliferation, migration and invasion in ccRCC.

### miR-363 suppresses xenograft tumour growth in vivo by targeting S1PR1

**Fig. 6:**
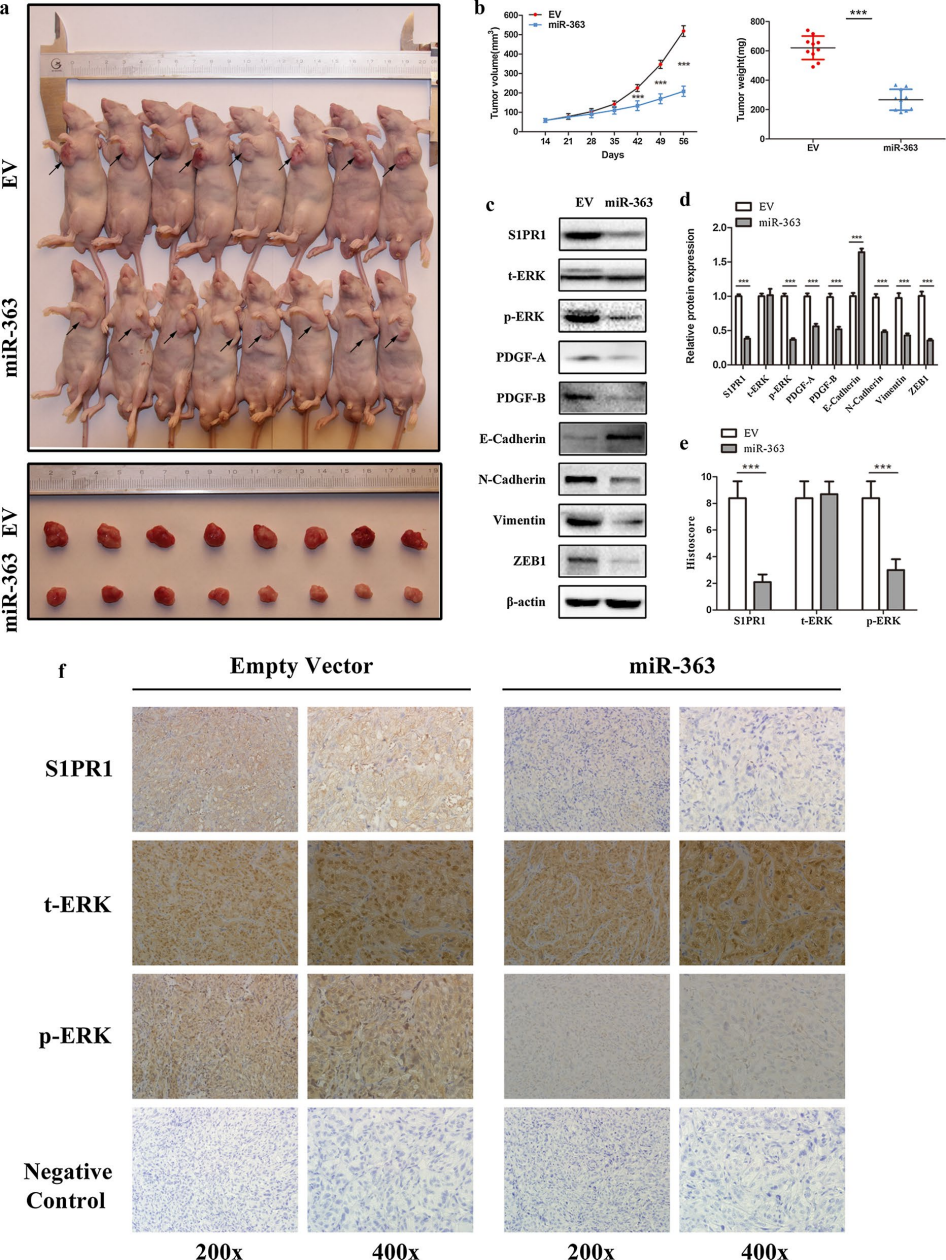
Overexpression of miR-363 suppresses xenograft tumour growth in vivo. **a** Xenograft tumours were obtained and dissected 8 weeks after subcutaneous injection of 786O cells stably transfected with lentiviral miR-363 particles or empty vector. **b** Comparison of tumour volume and weight between the miR-363 overexpression group and the EV group (10 mice per group). **c**, **d** Alterations in the protein levels of S1PR1, ERK and downstream genes of ERK in xenograft tumours between the miR-363 overexpression group and the EV group. **e** Histoscores of S1PR1, t-ERK and p-ERK in IHC-stained xenograft tumours between the miR-363 overexpression group and the EV group. **f** Representative IHC staining images of S1PR1, t-ERK, p-ERK and negative control in xenograft tumours between the miR-363 overexpression group and EV group. Data are presented as the mean ± SD. (*P < 0.05, **P < 0.01, ***P < 0.001)

To further explore whether miR-363 can affect the tumour growth of ccRCC cells in vivo, 786O cells stably transfected with lentiviral miR-363 particles or empty vector (EV) were subcutaneously injected into the left armpits of nude mice to establish the xenograft tumour model. All mice were sacrificed and dissected to obtain tumours 8 weeks after injection. The results showed that miR-363 overexpression in 786O cells markedly repressed tumour growth and significantly decreased tumour size compared to that in the EV group (Fig. 6a, b). Then, the mouse tumours were used for western blot and immunohistochemistry analysis. Similar results were obtained in vivo compared to that in vitro by western blot experiments. We found that miR-363 overexpression significantly suppressed the expression of S1PR1 and dramatically reduced the phosphorylation but not the total levels of ERK in vivo (Fig. 6c, d). We further examined downstream genes of ERK in mouse tumours. The results showed that miR-363 overexpression dramatically decreased the expression of PDGF-A, PDGF-B, N-cadherin, vimentin and ZEB1 and increased the expression of E-cadherin in vivo (Fig. 6c, d). In immunohistochemistry analysis, the results also confirmed that the expression of S1PR1 and phosphorylation of ERK were decreased, whereas the protein expression of total ERK was almost constant in the miR-363 overexpression group and the EV group (Fig. 6e, f). Collectively, these results further demonstrated that miR-363 acts as a tumour suppressor of ccRCC in vivo by downregulating S1PR1 and blocking ERK-related pathways.

## Discussion

Recent studies have revealed that dysregulation of miRNAs is common in cancer and that miRNAs can function as oncogenes or tumour suppressers [24–28]. Genome-wide expression profiling of miRNAs using microarray analysis of tumour tissue and matched normal tissues has recognized hundreds of downregulated and upregulated miRNAs between two groups [29, 30]. The differential miRNA patterns identified reveal a solid basis for further study. In addition to our previous miRNA expression profiling results, the detection of downregulated miR-363 expression levels in many profiles has prompted us to validate the functions of this miRNA. In our study, we found that miR-363 was significantly downregulated in ccRCC tissues compared to adjacent normal tissues. Patients with higher TNM stage, higher T stage and higher Fuhrman grade had lower expression levels of miR-363. Moreover, decreased miR-363 expression was associated with poor prognosis in this group of patients. Similar to our findings, miR-363 was markedly downregulated in colorectal cancer tissues and was negatively associated with the advanced stage of colorectal cancer [31]; decreased miR-363 expression promoted metastasis via EMT in non-small-cell lung cancer [18]. These studies showed that the miR-363 expression pattern is tissue specific and that miR-363 could serve as a tumour suppressor in certain human cancers, including ccRCC.

Proliferation, migration and invasion are three main phenotypes in cancers and are closely correlated with the biological functions of cancer cells. Our study indicated that upregulated miR-363 expression suppressed the proliferation, migration and invasion ability of ccRCC cells, whereas downregulated miR-363 expression enhanced these aspects. The miR-363 target was predicted to explore the potential mechanisms underlying the tumour-suppressive function of miR-363 in ccRCC. We found that S1PR1 was upregulated in ccRCC tissues and that its expression was inversely correlated with that of miR-363. Moreover, upregulated miR-363 expression obviously decreased S1PR1 expression at both the mRNA and protein levels and vice versa. The correlation between S1PR1 and miR-363 has been well established. By using a luciferase reporter assay, we identified that S1PR1 is a direct target of miR-363 and that knockdown of S1PR1 can phenocopy the effect of miR-363 overexpression in ccRCC. Subsequently, we performed functional rescue experiments of S1PR1 in 786O and 769P cells. Restoration of S1PR1 expression reversed the suppressive effects of the miR-363 mimic on the proliferation, migration and invasion abilities of ccRCC cells. In contrast, S1PR1 siRNA reversed the promotive effects of the miR-363 inhibitor on the proliferation, migration and invasion abilities of ccRCC cells. These findings suggested that miR-363 inhibited the proliferation, migration and invasion of ccRCC cells by directly targeting S1PR1.

S1PR1, originally named EDG1 (endothelial differentiation gene 1), belongs to a family of five G-protein-coupled receptors (S1PR1-5) [32]. Previous studies have indicated that S1PR1 plays a pivotal role in several tumours [33–37]. S1PR1 can regulate many functions of cancer cells, including proliferation, survival, migration, morphogenesis and angiogenesis, by modulating various downstream genes [35, 38–40]. In our study, we found that with upregulated miR-363 expression, there were several alterations detected in the signal pathway associated with cell proliferation, migration and invasion. It has been reported that S1PR1 can stimulate Ras, which is a MAPKKK, in an inhibitory G protein-dependent manner [41]. Activation of Ras can stimulate ERK, and ERK is an important MAPK that phosphorylates downstream transcription factors related to cell proliferation-, migration- and invasion-related genes, such as PDGF-A, PDGF-B and EMT-related genes [32, 42]. PDGF-A and PDGF-B are crucial signalling factors related to tumour progression by accelerating the proliferation of tumour cells [32]. The EMT process is generally considered an important mechanism of cancer malignancy and metastasis. It can transform the epithelial state of cancer cells to confer mesenchymal characteristics promoting the migration and invasion of cancer cells, resulting in metastasis [43]. In our study, we revealed that upregulated miR-363 remarkably decreased the phosphorylation but not the total levels of ERK in vitro, and these effects were reversed by overexpressing S1PR1 and vice versa. Moreover, downregulation of PDGF-A, PDGF-B, N-cadherin, vimentin and ZEB1 and upregulation of E-cadherin were associated with increased miR-363 levels, and these effects were reversed by overexpressing S1PR1 and vice versa. Further in vivo assays performed on a nude mouse model showed that miR-363 overexpression decreased xenograft tumour volume and weight. Similar to our findings in vitro, the in vivo assay revealed that upregulated miR-363 obviously suppressed the expression of S1PR1, significantly decreased the phosphorylation but not the total levels of ERK, and then markedly decreased the expression of PDGF-A, PDGF-B, N-cadherin, vimentin and ZEB1 and increased the expression of E-cadherin. Thus, the existence of the miR-363/S1PR1/ERK pathway may mostly explain the biological inhibition of proliferation, migration and invasion of tumour cells by miR-363 in ccRCC.

## Conclusions

In conclusion, our study demonstrated that miR-363 serves as a novel tumour suppressor in ccRCC and that decreased miR-363 levels are correlated with poor prognosis in patients with ccRCC. miR-363 overexpression suppresses the malignant phenotype of ccRCC cells by directly targeting S1PR1 and further affecting its downstream genes. Therefore, the miR-363/S1PR1/ERK pathway may be considered a potential therapeutic target for patients with ccRCC.

## Supplementary information

**Additional file 1: Table S1.** Primer sequences for qRT-PCR.

**Additional file 2: Figure S1.** Quantitative analysis of S1PR1, ERK and downstream genes of ERK relative protein expression in 786O and 769P cells by western blot. **a** Alterations of S1PR1, ERK and downstream genes of ERK protein level in 786O cells transfected with miR-363 mimic (versus NC mimics) and lentiviral S1PR1 plasmids (versus empty vector). **b** Alterations of S1PR1, ERK and downstream genes of ERK protein level in 769P cells transfected with miR-363 inhibitor (versus NC inhibitor) and siS1PR1 (versus siNC). Data are presented as the mean ± SD. (*P < 0.05, **P < 0.01, ***P < 0.001).

## Data Availability

The datasets used and analyzed in the current study are available from the corresponding author on reasonable request.

## Abbreviations

miRNAs: microRNAs
ccRCC: Clear cell renal cell carcinoma
DFS: Disease-free survival
EMT: Epithelial-mesenchymal transition
RCC: Renal cell carcinoma
3′-UTR: 3′-Untranslated region
PCR: Polymerase chain reaction
S1PR1: Sphingosine-1-phosphate receptor 1
S1P: Sphingosine-1-phosphate
ERK: Extracellular signal-regulated kinase
AJCC: American Joint Committee on Cancer
qRT-PCR: Quantitative real-time PCR
PPIA: Peptidylprolyl isomerase A
siRNA: Small interfering RNA
NC: Negative control
IHC: Immunohistochemistry
DAPI: 4′,6-diamidino-2-phenylindole
WT: Wild-type
MUT: Mutated
EV: Empty vector
SD: Standard deviation
HR: Hazard ratio
CI: Confidence interval
363M: miR-363 mimic
363I: miR-363 inhibitor
EDG1: The endothelial differentiation gene 1
