# Supplementary material for: miR-363 suppresses the proliferation, migration and invasion of clear cell renal cell carcinoma by downregulating S1PR1
Source: Cancer Cell Int. 2020 Jun 10;20:227. doi: 10.1186/s12935-020-01313-9 (PMC7288407; doi:10.1186/s12935-020-01313-9)
Supplement: Supplementary file 1 — Additional file 1: Table S1. Primer sequences for qRT-PCR. [file 12935_2020_1313_MOESM1_ESM.docx]

Table S1. Primer sequences for qRT-PCR

| Gene | Primer sequence (5′–3′) |
| --- | --- |
| miR-363 | Stem-loop RT primer: |
|  | CTCACAGTACGTTGGTATCCTTGTGATGTTTCGATGCCATATTGTACTGTGAGTACAGATG |
|  | Forward primer: ACACTCCAGCTGGGAATTGCACGGTATCCAT |
|  | Reverse primer: CTCACAGTACGTTGGTATCCTTGTG |
| U6 | Forward primer: CTCGCTTCGGCAGCACA |
|  | Reverse primer: AACGCTTCACGAATTTGCGT |
| S1PR1 | Forward primer: CAGCAAATCGGACAATTCCT |
|  | Reverse primer: GCCAGCGACCAAGTAAAGAG |
| PPIA | Forward primer: ATGGTCAACCCCACCGTGT |
|  | Reverse primer: TCTGCTGTCTTTGGGACCTTGTC |

qRT-PCR: quantitative real-time polymerase chain reaction; miR‐363: microRNA‐363; RT: reverse transcription; S1PR1: Sphingosine-1-phosphate receptor 1; PPIA: peptidylprolyl isomerase A.
