# Supplementary figures and images for: miR-363 suppresses the proliferation, migration and invasion of clear cell renal cell carcinoma by downregulating S1PR1
Source: Cancer Cell Int. 2020 Jun 10;20:227. doi: 10.1186/s12935-020-01313-9 (PMC7288407; doi:10.1186/s12935-020-01313-9)

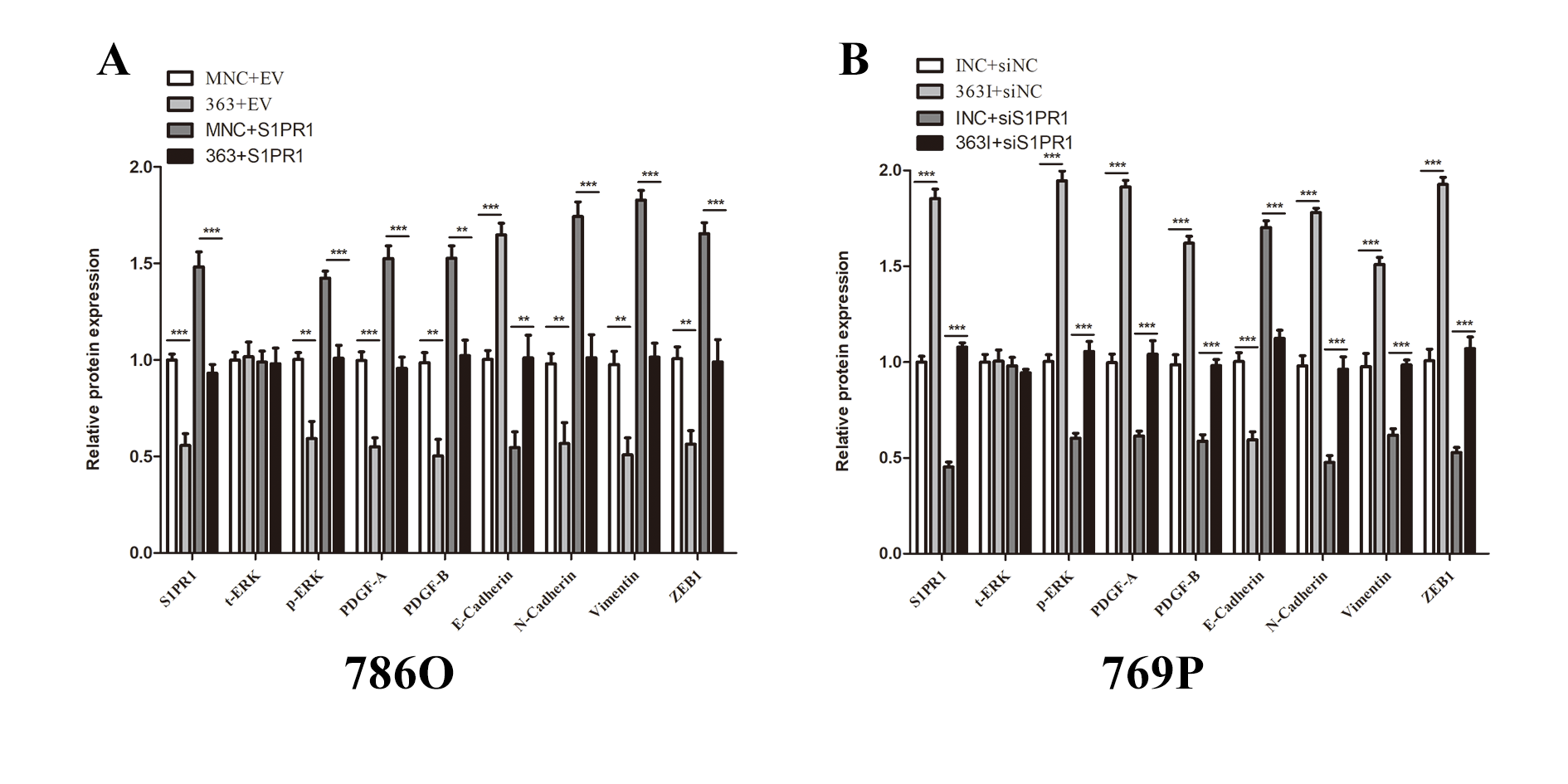

Supplement: Supplementary file 2 — Additional file 2: Figure S1. Quantitative analysis of S1PR1, ERK and downstream genes of ERK relative protein expression in 786O and 769P cells by western blot. a Alterations of S1PR1, ERK and downstream genes of ERK protein level in 786O cells transfected with miR-363 mimic (versus NC mimics) and lentiviral S1PR1 plasmids (versus empty vector). b Alterations of S1PR1, ERK and downstream genes of ERK protein level in 769P cells transfected with miR-363 inhibitor (versus NC inhibitor) and siS1PR1 (versus siNC). Data are presented as the mean ± SD. (*P < 0.05, **P < 0.01, ***P < 0.001). [file 12935_2020_1313_MOESM2_ESM.tif]
